# Beyond the blood-brain barrier: feasibility and technical validation of dual-compartment circulating tumor cells detection in high-grade glioma patients

**DOI:** 10.1007/s10143-025-03511-3

**Published:** 2025-04-11

**Authors:** Yu-Chung Juan, XianXiu Chen, Ju-Yu Tseng, Hui-Ju Lin, Cheng-Hao Hung, Po-Ren Hsueh, Jung-Ju Lin, Der-Yang Cho, Chun-Chung Chen

**Affiliations:** 1 Department of Neurosurgery, China Medical University Hospital, Taichung, Taiwan; 2 Neuroscience and Brain Disease Center, China Medical University, Taichung, Taiwan; 3 Graduate Institute of Biomedical Sciences, China Medical University, Taichung, Taiwan; 4 MiCareo Taiwan Co., Ltd, Taipei, Taiwan; 5 Department of Laboratory Medicine, China Medical University and Hospital, Taichung, Taiwan; 6 Department of Infectious Diseases, Department of Internal Medicine, China Medical University Hospital, China Medical University, Taichung, Taiwan; 7 Sleep Medicine Center, China Medical University Hospital, Taichung, Taiwan; 8 Graduate Institute of Acupuncture Science, China Medical University, Taichung, Taiwan

**Keywords:** Circulating tumor cells (CTCs), High-grade glioma, Liquid biopsy, MiSelect R II system, Cerebrospinal fluid, Microfluidic technology, Biomarkers, Glioblastoma

## Abstract

The elusive nature of brain tumor progression, hidden behind the blood-brain barrier, presents significant challenges for treatment monitoring in high-grade gliomas. In this feasibility study, we evaluate a novel approach to tracking glioblastoma through liquid biopsy, assessing whether tumor cells leave detectable molecular footprints in both blood and cerebrospinal fluid (CSF). Using the MiSelect R II System with specialized microfluidic technology, we analyzed paired blood and CSF samples from six glioblastoma patients, revealing a striking presence of circulating tumor cells (CTCs)– with higher abundance in CSF, where detection rates reached 100% compared to 83.3% in blood. Our technical validation demonstrates the system’s capability to identify CTCs through multi-marker analysis (EGFR+/GFAP+/CD45-). Preliminary observations revealed higher CTC counts in CSF (median 15.5 cells/mL) compared to blood (median 3.0 cells/mL), with notable differences between compartments suggesting they may reflect distinct aspects of disease biology. In a patient who developed progressive disease, we observed a substantial increase in CSF CTCs from 14 to 116 cells/mL, warranting further investigation in larger cohorts. Additionally, we detected CTC clusters in both compartments, an intriguing finding with potential biological significance. While our interim analysis provides technical proof-of-concept for CTC detection in glioblastoma patients, the limited sample size precludes definitive conclusions regarding clinical utility. These findings establish a methodological foundation for future comprehensive studies exploring the relationship between CTC dynamics and clinical outcomes in high-grade gliomas.

## Introduction

Glioblastoma (GBM) and other high-grade gliomas remain among the most challenging malignancies to treat [1, 2], with poor survival rates despite aggressive multimodal therapy [3]. The current standard of care, including surgical resection followed by radiation and chemotherapy [4], yields a median survival of only 12–15 months for GBM patients [5]. A major challenge in managing these aggressive tumors is the difficulty in detecting early recurrence [6] and distinguishing true progression from pseudoprogression on conventional imaging [7].

The blood-brain barrier (BBB) has traditionally been considered a significant obstacle in brain tumor diagnosis and monitoring, potentially limiting the utility of conventional liquid biopsy approaches in central nervous system malignancies [8]. However, recent evidence suggests that circulating tumor cells (CTCs) can be detected in the cerebrospinal fluid (CSF) and, to a lesser extent, in the peripheral blood of patients with primary brain tumors [9, 10]. This finding has opened new possibilities for monitoring disease status through minimally invasive blood sampling and lumbar punctures as complementary approaches to conventional imaging.

Several studies have demonstrated the presence of CTCs in both CSF and blood samples from glioma patients, with detection rates varying significantly based on the methodology used [11, 12]. Previous research has shown detection rates ranging from 20.6 to 77% using various techniques, including density gradient centrifugation, CTC-iCHIP technology, and matrix separation [13–17]. Notably, studies have found that CSF is often enriched for tumor cells compared to peripheral blood in primary CNS cancers, likely due to the BBB’s role in limiting systemic dissemination [18, 19]. This compartmentalization of tumor cells highlights the potential value of analyzing both blood and CSF for comprehensive liquid biopsy approaches in brain tumors.

The emergence of advanced microfluidic technologies has enhanced CTC detection capabilities. The MiSelect R II System, combined with specialized microfluidic chips and immunofluorescence techniques, offers a promising approach for detecting and characterizing CTCs in both blood and CSF samples. This system’s ability to identify CTCs based on multiple markers, including EGFR and GFAP expression, while excluding CD45-positive cells, provides a more comprehensive approach to CTC detection in brain tumors compared to single-marker methods [20].

Despite these technological advances, significant technical and biological challenges remain in establishing the feasibility of CTCs as biomarkers for brain tumor monitoring. The relationship between CTC presence and tumor recurrence needs careful investigation, particularly in the context of high-grade gliomas. Additionally, the potential for CTC analysis to complement imaging in differentiating true progression from pseudoprogression, a common challenge in neuro-oncology, requires technical validation before clinical implementation.

In this interim feasibility analysis of our prospective study, we evaluate the technical performance of the MiSelect R II System for detecting and characterizing CTCs in paired blood and CSF samples from patients with high-grade gliomas. We present preliminary observations on CTC detection rates in both compartments and provide initial insights into potential patterns of CTC counts in relation to disease status assessment. The primary focus of this analysis is to demonstrate the technical feasibility of detecting and characterizing CTCs in glioblastoma patients using this novel approach, while acknowledging that the limited sample size precludes definitive conclusions about clinical utility. By establishing the technical capabilities of this system in detecting glioma-derived CTCs, we aim to lay the groundwork for larger validation studies that will be necessary to determine the clinical significance of these preliminary observations.

This interim analysis addresses several key questions: (1) Can CTCs be reliably detected in both blood and CSF from high-grade glioma patients using the MiSelect R II System? (2) How do detection rates compare between blood and CSF samples? (3) What preliminary patterns can be observed regarding CTC counts across multiple time points? By focusing on these methodological aspects while presenting our initial observations within their appropriate technical context, we aim to advance the field’s understanding of the feasibility and technical considerations of liquid biopsy approaches in high-grade gliomas.

## Materials & methods

### Study design and patient population

This prospective, single-center feasibility study was conducted at China Medical University Hospital (CMUH) from September 2023. The study protocol was approved by the Institutional Review Board of CMUH (IRB/REC number: CMUH112-REC2-103) and conducted in accordance with the Declaration of Helsinki. All participants provided written informed consent.

### Patient selection

Six patients with newly diagnosed high-grade gliomas were enrolled in this interim analysis from a larger planned cohort. Eligibility criteria included: (1) age ≥ 20 years; (2) histologically confirmed diagnosis of high-grade glioma (WHO grade III or IV); and (3) ability to comply with study procedures and follow-up visits. Exclusion criteria comprised: (1) history of other active malignancies; (2) contraindications to lumbar puncture; (3) severe neurological impairment precluding informed consent; (4) significant intracranial hypertension; and (5) acute spinal trauma or high cervical tumors. All enrolled patients were diagnosed with IDH-wildtype glioblastoma (WHO grade IV) as detailed in Table 1.

**Table 1:** Demographic and baseline characteristics of glioblastoma patients in the interim analysis (*N* = 6) this table presents the demographic information, tumor characteristics, baseline CTC counts, and disease status of the six glioblastoma patients included in this interim analysis. The data illustrates the distribution of patient age, sex, tumor location, treatment history, and CTC detection in both blood and CSF samples. The presence of CTC clusters, an important finding in this study, is also documented

Characteristic	Value (*N* = 6)
**Age, years**	
Mean ± SD	52.3 ± 13.5
Median (range)	49.0 (38–70)
**Sex**,** n (%)**	
Male	3 (50.0)
Female	3 (50.0)
**Tumor Characteristics**	
**Tumor Type**,** n (%)**	
Glioblastoma, IDH-wild type (WHO grade IV)	6 (100.0)
**Tumor Location**,** n (%)**	
Temporal lobe	2 (33.3)
Parietal lobe	2 (33.3)
Frontal lobe	1 (16.7)
Multiple regions	1 (16.7)
**Treatment History**,** n (%)**	
Temozolomide	6 (100.0)
Bevacizumab	6 (100.0)
**Baseline CTC Counts**	
**Blood CTC count**	
Mean ± SD	3.5 ± 3.3
Median (range)	3.0 (0–9)
**CSF CTC count**	
Mean ± SD	18.7 ± 17.8
Median (range)	15.5 (4–52)
**Disease Status at Second Assessment**,** n (%)**	
Stable Disease	4 (66.7)
Partial Response	1 (16.7)
Progressive Disease	1 (16.7)
**CTC Clusters Observed**,** n (%)**	
In blood samples	1 (16.7)
In CSF samples	1 (16.7)

Abbreviations: SD, standard deviation; CTC, circulating tumor cell; CSF, cerebrospinal fluid; WHO, World Health Organization; IDH, isocitrate dehydrogenase

Note: Multiple regions refers to involvement of multiple anatomical areas (e.g., hippocampus, thalamus, and parietoccipital lobe in one patient)

### Clinical procedures and sample collection

#### Baseline assessment

At enrollment, comprehensive clinical data were collected, including demographic information, tumor characteristics, and treatment history. Initial tumor tissue samples obtained during surgical resection underwent pathological examination with standard histological processing and diagnosis according to WHO 2021 criteria. All six patients were diagnosed with IDH-wildtype glioblastoma (WHO grade IV), as shown in Table 1. While molecular subtyping for MGMT methylation status was performed for clinical care, additional molecular analyses (such as comprehensive GBM subtyping) were not part of this interim feasibility analysis but are planned for future comprehensive analysis of the full cohort.

#### Sample collection schedule

Patients underwent scheduled evaluations at baseline (post-surgery) and at follow-up intervals. For this interim analysis, data from the first two to three collection timepoints were available for each patient. At each timepoint, the following procedures were performed:

CSF samples (10 mL) were collected via lumbar puncture using a standard technique with a 22G spinal needle after local anesthesia with 1% lidocaine. For patients with ventriculoperitoneal shunts, CSF was collected through the Ommaya reservoir when available. CSF collection was performed by trained neurosurgeons under aseptic conditions. Samples were immediately transferred to sterile tubes containing EDTA as an anticoagulant.

Peripheral blood samples (10 mL) were collected in K2EDTA tubes by venipuncture following standard phlebotomy procedures. Blood draws were performed by trained nurses under aseptic conditions. Samples were gently inverted 8–10 times to ensure adequate mixing with the anticoagulant.

MRI scans were performed using standardized protocols on a 3T scanner (Siemens Magnetom Skyra, Erlangen, Germany). The imaging protocol included T1-weighted pre- and post-contrast sequences, T2-weighted, FLAIR sequences, diffusion-weighted imaging, and spectroscopy when clinically indicated. All scans were performed using the same scanner and protocol to ensure consistency.

Clinical assessments were conducted by the treating neurosurgeon and neuro-oncologist to evaluate disease status, neurological function, and performance status at each timepoint.

## Laboratory methods

### Sample processing and CTC analysis

#### Blood sample processing

Fresh blood samples were processed within 24 h of collection. The processing was performed according to the following protocol: samples underwent selective enrichment using the MiSelect R II System (MiCareo Inc., Taiwan) with SelectChip Rapid Dual microfluidic chip according to the manufacturer’s instructions. SelectSort EGFR antibody reagent was applied for 60 min at room temperature. Samples were processed according to standardized protocols including centrifugation at 800 xg for 10 min, washing steps with ISOTON^®^ II Diluent, and final sample preparation in 4–8 mL volume for analysis.

#### CSF sample processing

CSF samples were processed within 48 h of collection using the following protocol: initial centrifugation at 1500 rpm for 5 min to remove cellular debris, cell viability assessment using Trypan Blue staining, filtration through 100 μm mesh to remove large aggregates, and cell concentration adjustment to 1.25 × 10^5 cells/mL for optimal detection sensitivity. Positive controls using cell lines expressing EGFR and GFAP (U87 glioblastoma cell line) and negative controls with CD45 + leukocytes from healthy donors were processed in parallel to validate the detection system.

### CTC detection and characterization

#### MiSelect R II system protocol

The MiSelect R II System was operated using standardized parameters: DAPI (LED Power 30%, Exposure 0.02s), PE-EGFR (LED Power 100%, Exposure 1.5s), FITC-GFAP (LED Power 100%, Exposure 0.5s), and PerCP-CD45 (LED Power 45%, Exposure 0.5s). These parameters were established during system validation and maintained constant throughout the study to ensure consistency in CTC detection and enumeration.

#### CTC identification criteria

CTCs were identified based on the following stringent criteria: positive nuclear staining (DAPI+), EGFR expression (PE-EGFR+), GFAP expression (FITC-GFAP+), and absence of CD45 expression (PerCP-CD45-). Mean fluorescence intensity thresholds were set as follows: DAPI (1400–65535), EGFR (2300–65535), GFAP (10000–65535), and CD45 (500-30000). These thresholds were established based on validation studies with positive and negative control samples. Representative images of CTCs identified using these criteria are shown in Fig. 4.

CTC clusters were defined as aggregates of two or more CTCs that maintained cell-cell contact and traveled together in the circulation. The presence of CTC clusters was documented when observed in either blood or CSF samples, as reported in Table 1.

All CTC analyses were performed by trained laboratory technicians blinded to the clinical status of the patients. A second technician independently verified all positive CTC identifications to ensure accuracy.

### Clinical assessment and Follow-up

#### Disease status evaluation

Treatment response was assessed using the Response Assessment in Neuro-Oncology (RANO) criteria, which incorporate clinical and radiographic parameters. Disease status was categorized as:

Stable Disease: No new lesions and < 25% increase or < 50% decrease in enhancing tumor size compared to baseline, with stable or improved clinical status.

Partial Response: ≥50% decrease in enhancing tumor size compared to baseline with stable or improved clinical status and steroid use, maintained for at least 4 weeks, with no new lesions.

Progressive Disease: ≥25% increase in enhancing tumor size compared to baseline, or any new lesion, or definite clinical deterioration not attributable to other causes or to concurrent medication, or increasing steroid requirements.

MRI scans were evaluated by two independent neuroradiologists blinded to CTC results to minimize bias. In cases of discrepancy, a consensus was reached through discussion. Clinical assessments were performed by the treating neurosurgeon and neuro-oncologist at each follow-up visit.

### Safety monitoring

All adverse events were recorded and graded according to the Common Terminology Criteria for Adverse Events (CTCAE) version 5.0. Particular attention was paid to complications related to CSF sampling, including post-lumbar puncture headache, infection, bleeding, and neurological deterioration. Standard post-lumbar puncture care was provided to all patients, including hydration, lying flat for 2 h after the procedure, and monitoring for headache or neurological symptoms.

### Statistical analysis

#### Sample size calculation

While the planned sample size for the complete study was 24 patients (calculated to provide 80% power to detect a correlation coefficient of 0.5 between CTC counts and disease progression at a significance level of 0.05), this interim feasibility analysis focused on the first six enrolled patients to assess technical feasibility and generate preliminary observations, without formal hypothesis testing.

### Data analysis

Statistical analyses were performed using IBM SPSS Statistics version 25.0(IBM Corp., Armonk, NY, USA). Continuous variables were expressed as means ± standard deviation or medians with interquartile ranges as appropriate. Categorical variables were presented as frequencies and percentages. Due to the small sample size of this interim analysis, formal statistical testing was limited, and results are presented primarily as descriptive statistics.

**Fig. 1:**
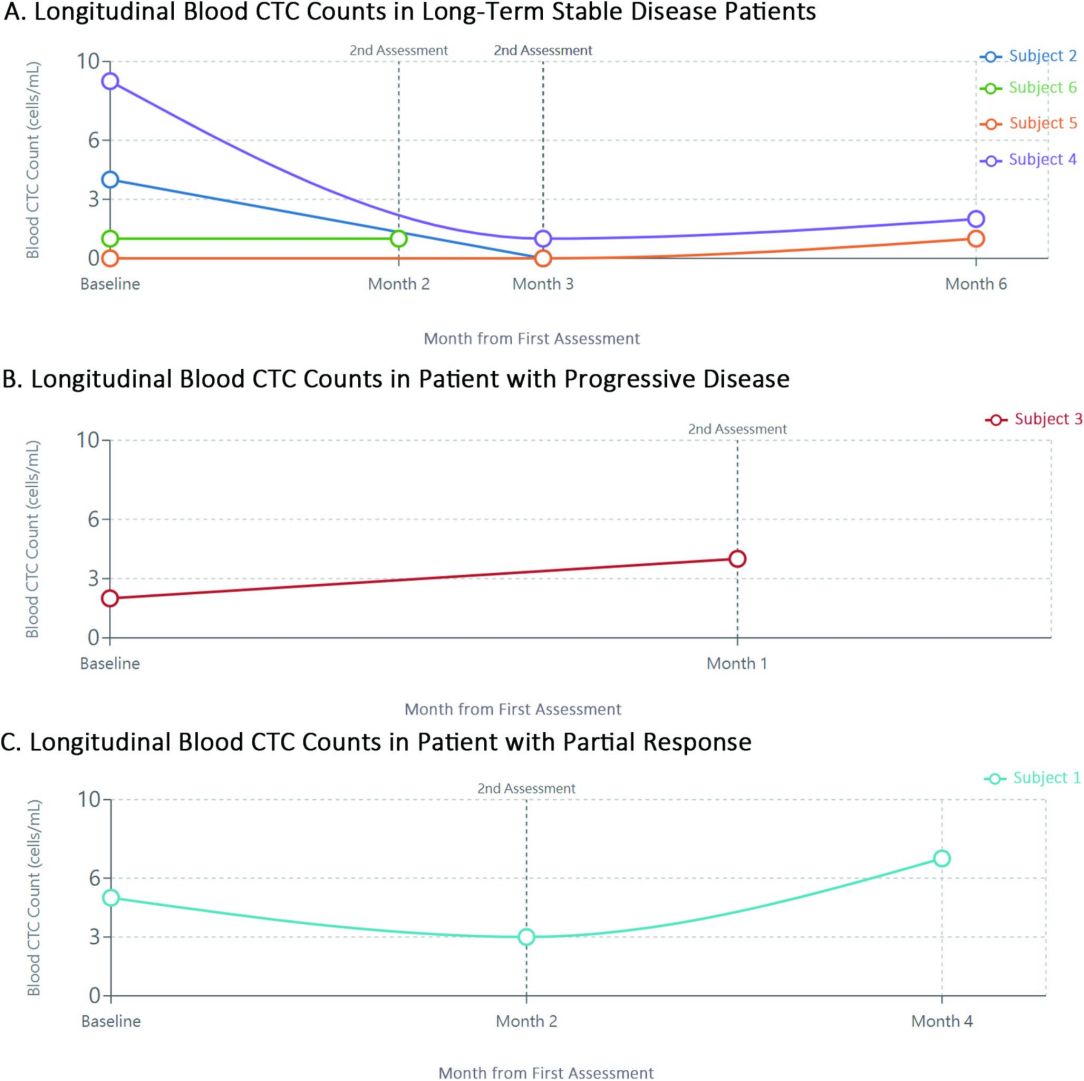
Longitudinal Analysis of Circulating Tumor Cells in Blood Samples from Glioblastoma Patients. Figure 1 shows the longitudinal monitoring of circulating tumor cell (CTC) counts in peripheral blood samples from six patients with IDH-wild type glioblastoma. Patients are grouped according to their clinical disease status at follow-up. (**A**) Blood CTC counts in patients with stable disease (*n* = 4), showing relatively consistent or decreasing counts over time. (**B**) Blood CTC dynamics in a patient with progressive disease (Subject 3), demonstrating an increase in CTC count from 2 to 4 cells/mL coinciding with clinical progression. (**C**) Blood CTC pattern in a patient with partial response followed by progression (Subject 1), showing an initial decrease followed by an increase in CTC count corresponding to disease progression. These preliminary findings suggest potential utility of blood CTC monitoring as a complementary approach to conventional imaging in assessing treatment response. CTC counts are presented as cells per mL of blood. Time points represent baseline (post-surgery) and follow-up assessments. Dotted vertical lines indicate clinical assessment timepoints. Error bars are not included as measurements represent single determinations at each timepoint

**Fig. 2:**
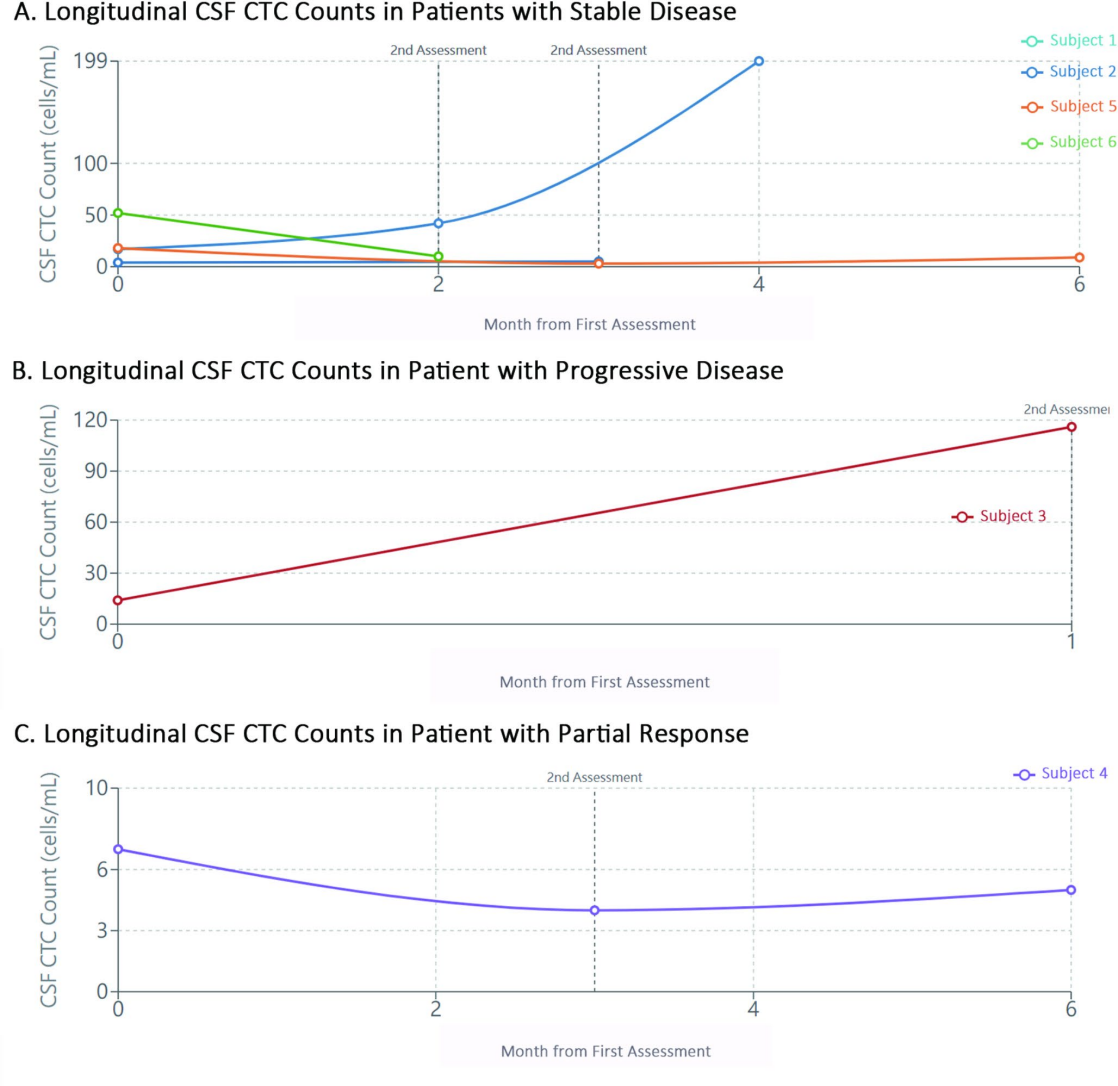
Longitudinal Analysis of Circulating Tumor Cells in Cerebrospinal Fluid Samples from Glioblastoma Patients. Figure 2 illustrates the longitudinal monitoring of circulating tumor cell (CTC) counts in cerebrospinal fluid (CSF) samples from the same six patients with IDH-wild type glioblastoma. (**A**) CSF CTC dynamics in patients with stable disease (*n* = 4), showing relatively consistent counts over the observation period. (**B**) CSF CTC pattern in a patient with progressive disease (Subject 3), demonstrating a substantial increase from 14 to 116 cells/mL, preceding clinical evidence of disease progression. (**C**) CSF CTC counts in a patient with partial response followed by progression (Subject 1), showing a pattern of increase corresponding to disease status changes. These preliminary observations suggest that CSF CTCs may be a more sensitive indicator of disease status compared to blood CTCs in high-grade glioma patients. CTC counts are presented as cells per mL of CSF. Time points represent baseline (post-surgery) and follow-up assessments. Dotted vertical lines indicate clinical assessment timepoints. Error bars are not included as measurements represent single determinations at each timepoint

**Fig. 3:**
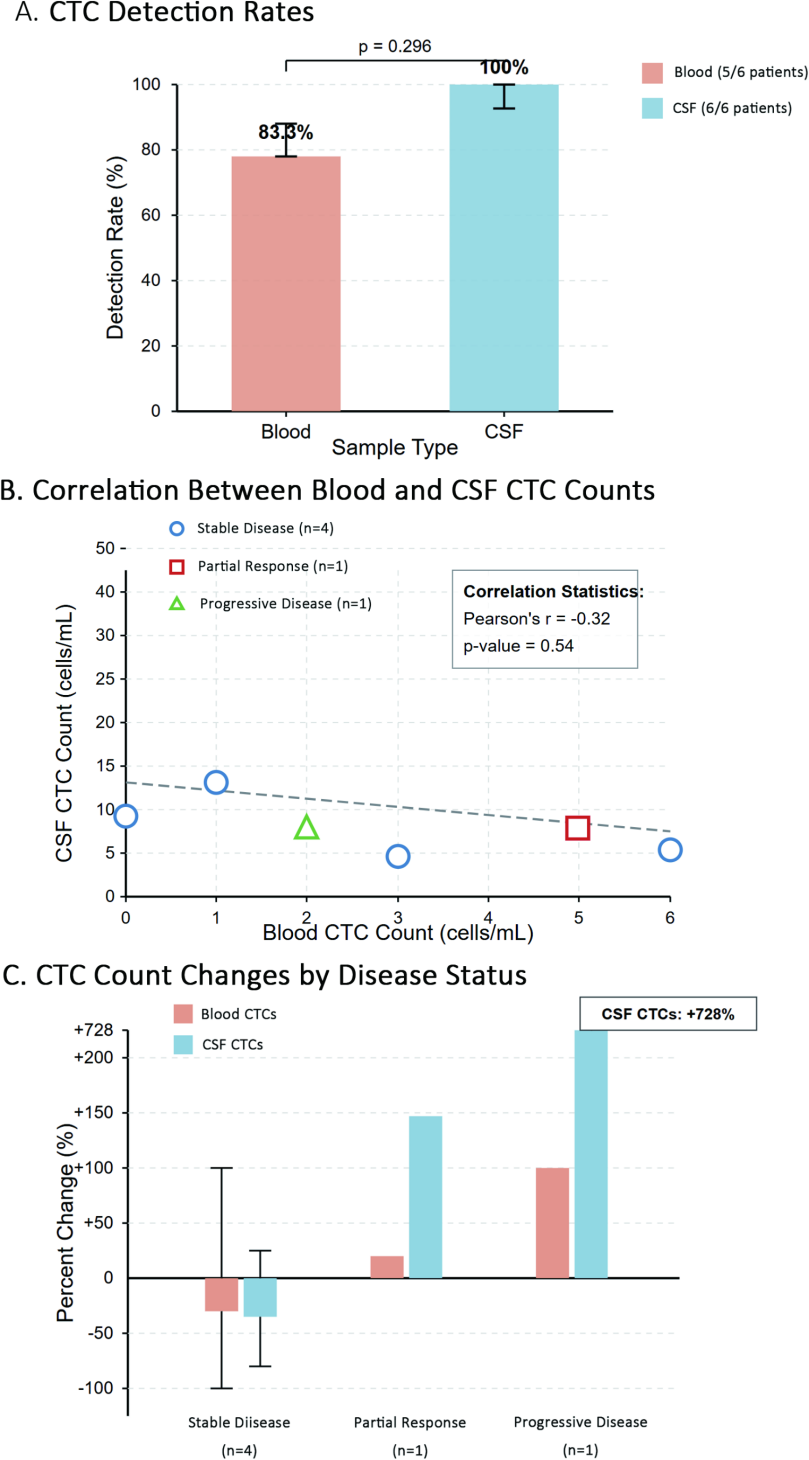
Comparison of CTC Detection in Blood and CSF Samples and Relationship to Disease Status. Figure 3 presents a comprehensive analysis of circulating tumor cell (CTC) detection in matched blood and CSF samples from glioblastoma patients. (**A**) Detection rates of CTCs in blood versus CSF samples across all patients (*n* = 6), demonstrating the higher sensitivity of CSF sampling (100%) compared to blood sampling (83.3%). (**B**) Correlation analysis between blood and CSF CTC counts at baseline assessment, showing a negative correlation (*r* = -0.525) that suggests these two compartments may reflect different aspects of disease biology. (**C**) Percent change in CTC counts between baseline and second assessment grouped by disease status, highlighting the substantial increase in CSF CTC count in the progressive disease case compared to stable disease and partial response cases. These findings support the potential complementary role of dual-compartment CTC monitoring in assessing treatment response in glioblastoma patients. In Panel A, detection rates were compared using Fisher’s exact test with 95% confidence intervals calculated using Wilson methods. In Panel B, different symbols represent disease status at second assessment. In Panel C, error bars represent standard error of the mean; for Progressive Disease and Partial Response (*n* = 1 each), no error bars are shown as these represent single patient observations

For visualization purposes, CTC counts in blood and CSF samples were plotted against time for each patient, grouped according to their disease status as determined by RANO criteria (Figs. 1 and 2). The relationship between blood and CSF CTC counts was explored through correlation analysis and visual examination (Fig. 3). Changes in CTC counts between baseline and follow-up assessments were calculated and presented in relation to disease status (Fig. 3).

**Fig. 4:**
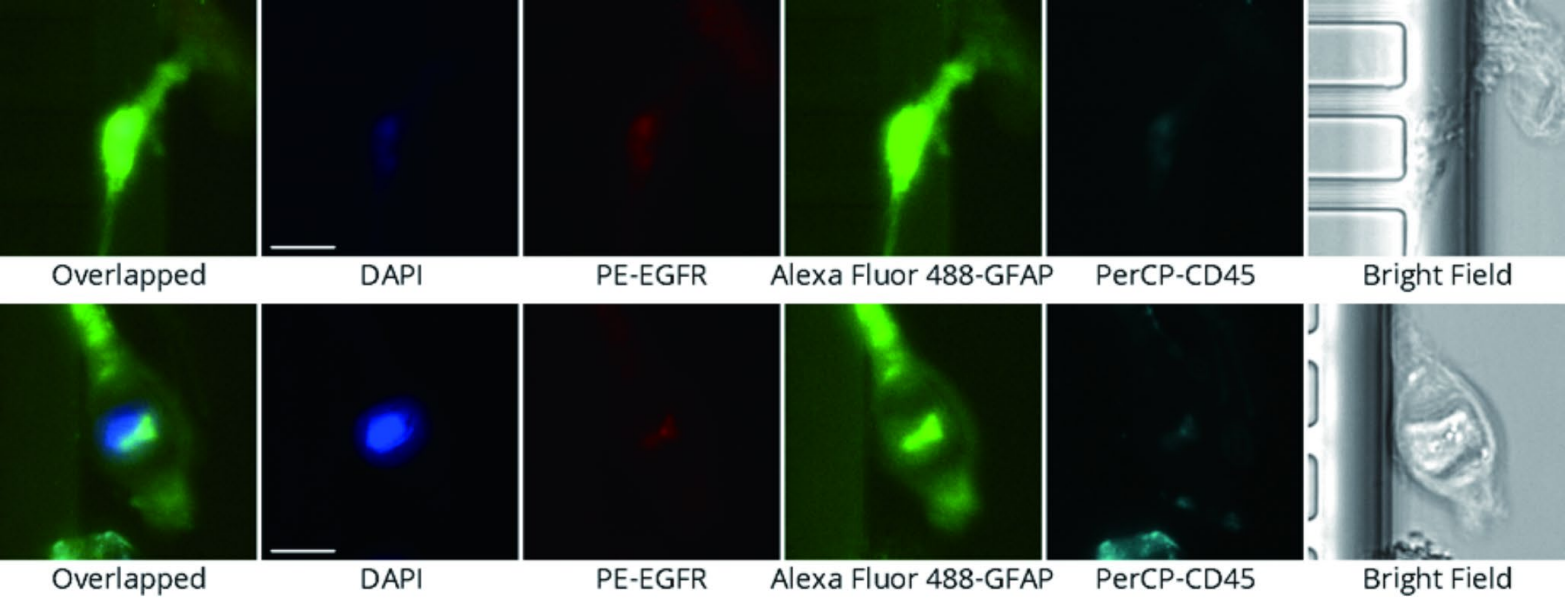
Identification of Circulating Tumor Cells in High-Grade Glioma Patients Using Multi-Marker Immunofluorescence. Figure 4 demonstrates the identification and characterization of circulating tumor cells (CTCs) in cerebrospinal fluid from a high-grade glioma patient using the MiSelect R II System. Representative immunofluorescence images show a CTC identified based on multiple marker expression: EGFR positivity (red), GFAP positivity (green), nuclear staining with DAPI (blue), and absence of the leukocyte marker CD45. The bright field image reveals the morphological features of the isolated CTC and CTC cluster. This multi-parametric approach enables reliable identification of CTCs originating from high-grade gliomas, distinguishing them from other cellular components in the CSF. CTCs were identified based on the following criteria: EGFR+ (red), GFAP+ (green), DAPI + nuclear staining (blue), and CD45- (absence of leukocyte marker). Scale bar represents 10 μm. Images were acquired using the MiSelect R II System with standardized exposure parameters. CTC cluster showed the same immunophenotypes with CTCs and defined as aggregates of two or more CTCs

### Interim analysis

This pre-planned interim analysis was conducted after the enrollment of the first six patients to assess technical feasibility and generate preliminary observations regarding CTC detection in high-grade glioma patients. As a feasibility assessment, emphasis was placed on detection rates, technical performance of the system, and initial observations of patterns rather than definitive statistical analysis of clinical correlations.

### Ethical considerations

The study was conducted in compliance with Good Clinical Practice guidelines and the Declaration of Helsinki. Patient confidentiality was maintained through coded identification numbers. All samples were processed and stored according to institutional biosafety protocols. The trial is registered at ClinicalTrials.gov (registration number pending). The study protocol, informed consent documents, and all amendments were approved by the Institutional Review Board of China Medical University Hospital before implementation.

## Results

### Patient characteristics

Between September 2023 and June 2024, six patients with newly diagnosed glioblastoma were enrolled and analyzed in this interim analysis. The demographic and clinical characteristics of these patients are summarized in Table 1. The cohort had a mean age of 52.3 ± 13.5 years (range 38–70 years), with equal distribution between males and females (3 each, 50%). All patients were diagnosed with IDH-wild type glioblastoma (WHO grade IV). Tumor locations included temporal lobe (*n* = 2, 33.3%), parietal lobe (*n* = 2, 33.3%), frontal lobe (*n* = 1, 16.7%), and multiple regions (*n* = 1, 16.7%). All patients received standard treatment with temozolomide and bevacizumab as part of their clinical care.

### Baseline CTC detection

At baseline assessment, CTCs were successfully detected in both blood and CSF samples using the MiSelect R II System. Figure 4 illustrates the identification of CTCs through multiparameter immunofluorescence analysis, demonstrating cells meeting the defined criteria of DAPI+, EGFR+, GFAP+, and CD45- phenotype. Blood samples showed a mean CTC count of 3.5 ± 3.3 cells (median 3.0, range 0–9). CSF samples demonstrated higher CTC counts with a mean of 18.7 ± 17.8 cells (median 15.5, range 4–52). The detection rate for CTCs was 83.3% (5/6 patients) in blood samples and 100% (6/6 patients) in CSF samples at baseline, suggesting superior sensitivity of CSF sampling for CTC detection in glioblastoma patients.

### Sequential CTC analysis in blood samples

Sequential measurements of blood CTCs across multiple timepoints revealed different patterns based on disease status, as illustrated in Fig. 1. Patients with stable disease (*n* = 4) demonstrated relatively consistent or decreasing blood CTC counts across sequential measurements. Subject 2 showed counts of 4 cells at baseline and 0 cells at second assessment, while Subject 6 maintained consistent counts of 1 cell across two timepoints. Subjects 4 and 5 similarly showed stable or gradually decreasing counts across three timepoints, with Subject 4 decreasing from 9 to 1 to 2 cells and Subject 5 maintaining low counts of 0, 0, and 1 cells.

The patient who developed progressive disease (Subject 3) showed an increase in blood CTC counts from 2 cells at baseline to 4 cells at the second timepoint, corresponding with clinical progression. One patient (Subject 1) showed an initial decrease in blood CTC count from 5 to 3 cells, followed by an increase to 7 cells at the third timepoint, which coincided with the transition from stable disease to progressive disease.

These preliminary observations suggest the potential for blood CTC monitoring to reflect disease status changes, although the limited sample size precludes definitive conclusions about the predictive value of these patterns.

### Sequential CTC analysis in CSF samples

Analysis of CSF CTC counts across sequential timepoints revealed more pronounced changes compared to blood samples, as shown in Fig. 2. Patients with stable disease showed varying patterns, but generally maintained lower CTC counts relative to patients with progressive disease. Among the stable disease patients, Subject 2 maintained relatively consistent CSF CTC counts (4 to 5 cells), while Subject 6 showed a decrease from 52 to 10 cells. Subjects 4 and 5 demonstrated initial decreases followed by slight increases, with Subject 4 changing from 7 to 4 to 5 cells and Subject 5 from 18 to 3 to 9 cells.

Notably, the patient who developed progressive disease (Subject 3) exhibited a striking increase in CSF CTC counts from 14 cells at baseline to 116 cells at the second timepoint, representing an approximately 8-fold increase concurrent with clinical progression. Similarly, Subject 1, who initially had stable disease but later progressed, showed a progressive increase in CSF CTC counts from 17 to 42 to 199 cells across three timepoints, with the most substantial increase corresponding to the transition to progressive disease.

These observations suggest that CSF CTC counts may demonstrate more pronounced changes in relation to disease status compared to blood CTC counts, potentially offering greater sensitivity for detecting disease progression. However, these findings represent preliminary observations requiring validation in larger cohorts.

### Comparison between blood and CSF CTC detection

Comparative analysis of CTC detection in blood versus CSF revealed several noteworthy patterns, as illustrated in Fig. 3. Detection rates were higher in CSF (100%) compared to blood (83.3%), suggesting that CSF may provide superior access to tumor-derived cells in glioblastoma patients. Furthermore, CSF consistently yielded higher absolute CTC counts compared to matched blood samples (median 15.5 versus 3.0 cells at baseline).

Interestingly, correlation analysis between blood and CSF CTC counts at baseline revealed a negative correlation (*r* = -0.525), suggesting that these two compartments may reflect different aspects of disease biology rather than directly mirroring each other. This observation highlights the potential complementary value of dual-compartment sampling for comprehensive CTC analysis in brain tumors.

Analysis of percent changes in CTC counts between baseline and second assessment showed more pronounced changes in CSF compared to blood across all disease status categories. As shown in Fig. 3C, the progressive disease case demonstrated a substantially larger percent increase in CSF CTCs (700%) compared to blood CTCs (100%). Similarly, patients with stable disease showed more modest changes in CSF CTC counts compared to the progressive disease case, further supporting the potential biological relevance of CSF CTC dynamics in relation to disease status.

### CTC clusters and morphological observations

An important observation in this feasibility study was the detection of CTC clusters in both blood and CSF samples during follow-up assessments. CTC clusters, defined as aggregates of two or more CTCs maintaining cell-cell contact, were detected in blood samples from Subject 1 (second assessment) and in CSF samples from Subject 4 (second assessment). Representative images of CTCs, including both single cells and clusters, were captured using the MiSelect R II System as shown in Fig. 4.

Morphological assessment of detected CTCs revealed cells with high nuclear-to-cytoplasmic ratios, irregular nuclear contours, and prominent nucleoli, consistent with the cytological features of high-grade glioma cells. CTCs displayed strong EGFR and GFAP expression, with varying intensity patterns observed between patients and between single CTCs and CTC clusters. These preliminary morphological observations suggest that CTC analysis may potentially provide insights into tumor cell characteristics beyond simple enumeration, though detailed phenotypic characterization was beyond the scope of this interim analysis.

### Disease status assessment and clinical correlation

At second assessment, four patients (66.7%) showed stable disease, one patient (16.7%) demonstrated partial response, and one patient (16.7%) exhibited progressive disease based on RANO criteria. This distribution allowed for preliminary observations regarding CTC patterns across different clinical scenarios, though the limited number of cases in each category precludes definitive conclusions about predictive value.

Patients with stable disease generally maintained relatively stable or decreasing CTC counts, particularly in CSF samples. The patient with progressive disease demonstrated marked increases in both blood and CSF CTC counts, with the increase in CSF being particularly pronounced. These preliminary observations suggest potential correlation between CTC dynamics and disease status, though validation in larger cohorts is essential to establish clinical utility.

### Safety outcomes

All CSF and blood sampling procedures were completed successfully without any grade 3 or higher adverse events. Minor adverse events included mild post-lumbar puncture headache in two patients (33.3%), which resolved with conservative management including hydration and analgesics. No complications related to CSF sampling such as infection, bleeding, or neurological deterioration were reported during the study period, supporting the feasibility and safety of serial CSF sampling in this patient population.

## Discussion

This interim analysis of our prospective study provides several important insights into the technical feasibility of CTC detection in high-grade gliomas using the MiSelect R II System. Our findings demonstrate the capability of detecting CTCs in both blood and CSF samples from glioblastoma patients, with notably higher detection rates and CTC counts in CSF compared to blood samples. These results align with previous studies suggesting that CSF may be a more reliable source for liquid biopsy in primary brain tumors due to the restrictive nature of the blood-brain barrier.

The consistently higher CTC counts in CSF (median 15.5 / 8 mL, range 4–52) compared to blood samples (median 3.0 / 8 mL, range 0–9) at baseline support the emerging consensus that CSF provides superior access to tumor-derived material in central nervous system malignancies. This observation is particularly relevant given the historical challenges in detecting CTCs in blood samples from glioma patients, where detection rates in previous studies have ranged from 20.6 to 77% using various technologies [13–17]. Our detection rate of 83.3% in blood samples and 100% in CSF samples suggests that the MiSelect R II System, with its specialized microfluidic chip design and multi-marker detection approach, may offer improved sensitivity compared to traditional CTC detection methods.

The sequential analysis of CTC counts revealed several noteworthy patterns that warrant further investigation. In patients with stable disease, blood CTC counts remained relatively constant, while CSF CTC counts showed more variability but generally remained within a lower range. Conversely, the patient who developed progressive disease demonstrated marked increases in CTC counts in both blood (2 to 4 cells) and particularly in CSF (14 to 116 cells). While these observations are limited by the small sample size of this interim analysis, they raise the possibility that dynamic changes in CTC counts, particularly in CSF, might reflect underlying disease status.

Of particular interest is the observation of CTC clusters in both blood and CSF samples during follow-up assessments. The detection of these clusters, which have been associated with increased metastatic potential in other cancers, may provide additional insights into disease biology in glioblastoma patients. The presence of clusters in Subject 1’s blood sample and Subject 4’s CSF sample during the second assessment warrants further investigation into their clinical significance and potential role in disease progression.

Our study has several important limitations that must be acknowledged. First, the small sample size of this interim analysis (*n* = 6) significantly limits the statistical power and generalizability of our findings. Second, the relatively short follow-up period may not fully capture the long-term dynamics of CTC counts and their relationship to clinical outcomes. Third, while our study demonstrates the feasibility of detecting CTCs, the biological significance of different CTC levels and the optimal thresholds for clinical decision-making remain to be determined. Fourth, the molecular GBM subtyping and phenotypic characterization of CTCs through additional markers and FISH analysis were not performed in this interim analysis but are planned for subsequent research.

Several technical challenges emerged during our study that warrant consideration in future research. The processing of CSF samples required careful standardization to maintain cell viability, and the optimal timing of sample collection relative to treatment cycles needs further investigation. Additionally, while CTC clusters were observed, their detailed molecular characterization was beyond the scope of this interim analysis.

Our findings raise several important questions for future research. The relationship between CTC counts and molecular markers such as IDH mutation status and MGMT methylation warrants investigation in larger cohorts. The potential role of CTC analysis in predicting treatment response and distinguishing pseudoprogression from true progression needs prospective validation. Furthermore, the biological significance of CTC clusters and their potential role in disease dissemination requires detailed investigation.

These preliminary results suggest several potential clinical applications that merit further exploration. Regular monitoring of CSF CTCs might provide an early warning system for disease progression, potentially allowing for earlier intervention. The differential CTC counts between blood and CSF samples might help guide the choice of liquid biopsy source in future clinical applications. The detection of CTC clusters might offer additional prognostic information, though this requires validation in larger studies.

The integration of advanced technologies, such as artificial intelligence -based analysis and multi-omics approaches, could further enhance the sensitivity and specificity of CTC detection. Additionally, future studies should explore the mechanisms by which glioma cells breach the BBB and enter systemic circulation, as this knowledge could lead to new therapeutic targets for preventing disease progression.

As this study continues, we aim to address several key questions: the correlation between CTC dynamics and long-term clinical outcomes, the molecular characterization of detected CTCs and their potential heterogeneity, and the development of quantitative thresholds for clinical decision-making. By establishing the technical feasibility of detecting and characterizing CTCs in glioblastoma patients, this interim analysis provides a foundation for more comprehensive investigations into the clinical utility of liquid biopsy approaches in high-grade gliomas.

## Conclusion

This feasibility study demonstrates that circulating tumor cells can be successfully detected in both blood and cerebrospinal fluid from patients with high-grade gliomas using the MiSelect R II System with specialized microfluidic technology. Our interim analysis reveals several important technical and biological insights. First, CSF appears to be a more sensitive compartment for CTC detection in glioblastoma patients, with a 100% detection rate compared to 83.3% in blood samples. Second, the observed negative correlation between blood and CSF CTC counts suggests these compartments may reflect different aspects of disease biology, highlighting the potential complementary value of dual-compartment sampling. Third, the detection of CTC clusters in both blood and CSF represents an intriguing finding that warrants further investigation regarding their biological significance in high-grade gliomas.

The preliminary observations of CTC dynamics in relation to disease status—particularly the marked increase in CSF CTC counts in the patient with progressive disease—suggest potential utility for CTC monitoring as a complementary approach to conventional imaging. However, we emphasize that these findings represent initial observations from a limited sample size that require validation in larger cohorts before clinical application can be considered. The higher CTC counts consistently observed in CSF compared to blood align with the biological understanding of the blood-brain barrier’s role in limiting systemic dissemination of brain tumor cells.

From a methodological perspective, this study establishes the feasibility of the multi-marker detection approach (EGFR+/GFAP+/CD45-) for reliable identification of glioma-derived CTCs. The safety profile observed supports the feasibility of serial sampling in this patient population, an important consideration for longitudinal monitoring applications. As our understanding of liquid biopsy in neuro-oncology continues to evolve, this technical groundwork provides an essential foundation for more comprehensive studies investigating the clinical utility of CTC analysis.

Future research should address several key questions raised by this feasibility study: the relationship between CTC dynamics and long-term clinical outcomes, the molecular and phenotypic characterization of detected CTCs, and the establishment of quantitative thresholds for clinical decision-making. By advancing our technical capabilities for detecting and characterizing CTCs in high-grade glioma patients, we move closer to the goal of developing minimally invasive approaches for monitoring treatment response and detecting early disease progression in this challenging patient population.

## Data Availability

No datasets were generated or analysed during the current study.
